# Loss of Müller cell glutamine synthetase immunoreactivity is associated with neuronal changes in late-stage retinal degeneration

**DOI:** 10.3389/fnana.2023.997722

**Published:** 2023-03-07

**Authors:** Hallur Reynisson, Michael Kalloniatis, Erica L. Fletcher, Mohit N. Shivdasani, Lisa Nivison-Smith

**Affiliations:** ^1^School of Optometry and Vision Science, UNSW Sydney, Sydney, NSW, Australia; ^2^Graduate School of Biomedical Engineering, UNSW Sydney, Sydney, NSW, Australia; ^3^Faculty of Medicine (Optometry), Deakin University, Waurn Ponds, VIC, Australia; ^4^Department of Anatomy and Physiology, The University of Melbourne, Melbourne, VIC, Australia; ^5^Bionics and Bio-robotics, Tyree Foundation Institute of Health Engineering, Kensington, NSW, Australia

**Keywords:** Müller cells, photoreceptor degeneration, retinal remodeling, neurodegeneration, glutamine synthetase, rd1 mouse model

## Abstract

**Introduction:**

A hallmark of photoreceptor degenerations is progressive, aberrant remodeling of the surviving retinal neurons and glia following photoreceptor loss. The exact relationship between neurons and glia remodeling in this late stage of retinal degeneration, however, is unclear. This study assessed this by examining Müller cell dysfunction via glutamine synthetase immunoreactivity and its spatial association with retinal neuron subpopulations through various cell markers.

**Methods:**

Aged Rd1 mice retinae (P150 – P536, *n* = minimum 5 per age) and control heterozygous rd1 mice retinae (P536, *n* = 5) were isolated, fixed and cryosectioned. Fluorescent immunolabeling of glutamine synthetase was performed and retinal areas quantified as having low glutamine synthetase immunoreactivity if proportion of labeled pixels in an area was less than two standard deviations of the mean of the total retina. Other Müller cell markers such as Sox9 and Glial fibrillary acidic protein along with neuronal cell markers Calbindin, Calretinin, recoverin, Protein kinase C-α, Glutamic acid decarboxylase 67, and Islet-1 were then quantified within areas of low and normal synthetase immunoreactivity.

**Results:**

Glutamine synthetase immunoreactivity was lost as a function of age in the rd1 mouse retina (P150 – P536). Immunoreactivity of other Müller cell markers, however, were unaffected suggesting Müller cells were still present in these low glutamine synthetase immunoreactive regions. Glutamine synthetase immunoreactivity loss affected specific neuronal populations: Type 2, Type 8 cone, and rod bipolar cells, as well as AII amacrine cells based on reduced recoverin, protein kinase Ca and parvalbumin immunoreactivity, respectively. The number of cell nuclei within regions of low glutamine synthetase immunoreactivity was also reduced suggesting possible neuronal loss rather than reduced cell marker immunoreactivity.

**Conclusion:**

These findings further support a strong interplay between glia-neuronal alterations in late-stage degeneration and highlight a need for future studies and consideration in intervention development.

## 1. Introduction

Retinitis Pigmentosa (RP) is the most common inherited retinal degeneration affecting one in 4,000 individuals (Hartong et al., 2006). It results from the death of photoreceptors leading to significant visual impairment and ultimately, blindness. A large body of evidence shows that the impact of RP continues to the inner retina which undergoes change secondary to photoreceptor loss (Jones et al., 2003; Marc et al., 2003; Strettoi et al., 2003; Jones and Marc, 2005; Marc et al., 2007; Chua et al., 2009, 2013; Kalloniatis et al., 2013; Greferath et al., 2015; Pfeiffer et al., 2020b; Strettoi et al., 2022). These changes are complex, and include anatomical, metabolic and functional alterations of both neurons and glia in the inner retina (Jones et al., 2003; Marc et al., 2003; Strettoi et al., 2003; Jones and Marc, 2005; Marc et al., 2007; Chua et al., 2009, 2013; Kalloniatis et al., 2013; Greferath et al., 2015).

An added complexity is that inner retinal changes are dependent on disease stage. For example, Chua et al. (2009) found that neurochemical remodeling of ionotropic glutamate receptors on bipolar cells in the rd1 mouse was only evident during active cone degeneration and was lost following total photoreceptor death. Marc et al. (2003) noted that specific, significant anatomical changes to the inner retina such as neuronal migration, cell death and glial seal completion only occurred late in the course of disease, well beyond photoreceptor death (Jones et al., 2003). Considering that many vision restoration strategies are targeted to individuals with well-established vision loss, furthering understanding of late-stage degeneration is most relevant to the successful development and deployment of such interventions.

Glial remodeling also exhibits separate, time dependent phases of change. For example, early degeneration is associated with loss of Müller cell processes and hyperexpression of glial fibrillary acidic protein (GFAP) while late degeneration is associated with the converse, Müller cell hypertrophy and glial seal formation (Bringmann et al., 2006; Chua et al., 2013). Metabolic profile and protein expression of Müller cells also becomes distinctly “chaotic” in late-stage degeneration. Specifically, metabolic amino acid signatures between neighboring Müller cells are significantly heterogenous with no obvious explanation for variation (Jones et al., 2016; Pfeiffer et al., 2020a). Major metabolic enzymes, cellular retinaldehyde-binding protein CRALBP and glutamine synthetase also undergo varying levels of loss with the latter demonstrated within human RP retinae (Jones et al., 2016), the P347L rabbit retinae (Pfeiffer et al., 2016, Pfeiffer et al., 2020b) and the rd1-Fos-Tau-LacZ (rd1-FTL) mouse retinae (Greferath et al., 2015). Greferath et al. (2015) further postulated that inner retinal neurons within regions of abnormal glutamine synthetase immunolabeling were abnormal based on FTL expression suggesting a potential cause-and-effect relationship to explain neuronal and glial changes in late-stage retinal degeneration. Such a relationship could help predict the course of inner retinal change in late-stage degeneration and be of significant benefit in guiding intervention approaches.

A quantitative time course of Müller cell dysfunction (based on loss of glutamine synthetase immunoreactivity) is currently unknown. However, qualitatively data from Pfeiffer et al. (2020b) presents a potential degeneration dependent loss in the P347L rabbit. Similarly, investigation of the specific neuronal populations which are present within regions of Müller cell dysfunction is also limited. Thus, the aim of this study was to determine the time course of glutamine synthetase loss in Müller cells in late-stage retinal degeneration and the identity the neuronal cell types remaining within these regions of altered Müller cell function.

## 2. Materials and methods

### 2.1. Animals

Rd1 mice (on a C57Bl/6 background) (Farber et al., 1994) were studied at post-natal day P150 - P536 (*n* = minimum 5 per age). The control mice (C57Bl/6) were examined at the oldest age only (P536; *n* = 5). Animals were maintained on a 12 h light/dark cycle and had access to standard mouse chow and water *ad libitum*. The experimental protocols in this study were approved by the University of Melbourne and UNSW Sydney Animal Ethics committees.

### 2.2. Tissue fixation and immunolabeling

Mouse retinae were processed and immunostained as described previously (Nivison-Smith et al., 2013, 2014, 2015, 2017). Briefly, mice were killed by cervical dislocation. Eyes were enucleated immediately, and the anterior structures removed, under constant fluorescent room lighting, creating an eyecup preparation. Eyecups were then fixed for 30 min in 4% (w/v) paraformaldehyde and 0.01% (w/v) glutaraldehyde in 0.10 M phosphate buffer. Tissues were then washed in 0.10 M phosphate buffer before cryo-protection in graded 30% (w/v) sucrose and cryo-sectioned in the vertical plane at a thickness of 120 μm.

For immunostaining, retinal sections were blocked for 60 min with 6% (v/v) goat serum, 1% (w/v) bovine serum albumin, 0.1% (v/v) Triton-X then incubated overnight at 4^°^C with primary antibodies at the dilutions specified in Table 1. Primary antibodies were detected with anti-chicken AlexaFluor 488, anti-rabbit AlexaFluor 488 or 594, or anti-mouse AlexaFluor 405 or 488 (Thermo Fisher Scientific, Waltham, MA, USA). Sections were incubated with secondary antibodies at a 1:500 dilution for 2 h at room temperature. All antibody dilutions were made in 3% (v/v) goat serum, 1% (w/v) bovine serum albumin, 0.1% (v/v) Triton-X. In a subset of samples, counterstaining was performed with 2-(4-amidinophenyl)-1H -indole-6-carboxamidine (DAPI) diluted 1:1000 in MilliQ water. All samples were mounted in Citifluor mounting media (ProSciTech, QLD, Australia). Sections were imaged using an FV1200 Scanning Laser Microscope (Olympus Australia, Notting Hill, VIC, Australia).

**TABLE 1:** Details of the antibodies used in this study.

Antigen	Immunogen	Specificity*	Manufacturer, cat no.	Host	Dilution	Retinal cell types labeled	Length analysed (μm)
Calbindin	Purified bovine kidney calbindin-D-28K	–	Sigma-Aldrich; C9848	Ms; monoclonal	1:1000	Subpopulations of amacrine cells and horizontal cells	7,165
Calretinin (CR)	Rat calretinin, amino acids 38–151	The antibody binds to the Ca^2+^ binding protein Calretinin	BD Transduction; 610908	Ms; monoclonal	1:1000	Populations of amacrine and ganglion cells	24,555
Glial fibrillary acidic protein (GFAP)	GFAP from pig spinal cord	The antibody reacts specifically with GFAP in immunoblotting assays and labels astrocytes.	Sigma Aldrich; G3893	Ms; monoclonal	1:1000	Müller cell presence	6,698
Glutamic acid decarboxylase GAD67	Synthetic peptide from mouse GAD67 (amino acids 87–106	Reacts specifically with GAD67	Sigma-Aldrich; G5419	Ms; monoclonal	1:500	GABAergic amacrine cells	7,630
Glutamine synthetase (GS)	Glutathione conjugated to glutaraldehyde	–	Abcam; ab93439	Rb; polyclonal	1:500	Müller glia in terms of glutamate metabolism	191,030
Islet-1	Truncated rat islet protein corresponding to amino acids 178–349	–	Developmental Studies Hybridoma Bank; 39.4D5	Ms; monoclonal	1:200	ON bipolar cells	14,270
Parvalbumin (PV)	Frog muscle parvalbumin	Recognizes parvalbumin in a Ca^2+^ ion-dependent manner.	Sigma-Aldrich; P3088	Ms; monoclonal	1:500	Amacrine cells, specifically AII amacrine cells	11,860
Protein kinase C-α (PKCα)	Purified bovine brain PKC	Reacts with the 80 kDa polypeptide of PKC.	Sigma-Aldrich; P5704	Ms; monoclonal	1:400	Rod bipolar cells	35,620
Recoverin	Recombinant human recoverin	Recognizes recoverin.	Chemicon (Millipore); AB5585	Rb; polyclonal	1:1000	Cone bipolar cell type 2 and type 8	13,590
SOX9	C-terminal sequence of human Sox9	Recognizes Sox9	Chemicon (Millipore); AB5535	Rb; polyclonal	1:2000	Astrocytes and Müller cells	–

Rb, rabbit; Ms, mouse.

*Specificity retrieved from manufacturers websites.

All samples that were stained for the same antibody were processed at the same time with no difference in antibody batch number to address possible issues from histological preparation. To decrease effect of eccentricity all samples were taken within 500 μm of the central retina from temporal to nasal. Images were taken within a week of staining with fixed laser settings for DAPI, AlexaFluor488, and AlexaFluor594 labeling to decrease errors due to laser variability.

### 2.3. Immunolabeling quantification

#### 2.3.1. Sliding window and pixel threshold criteria

A systematic approach was developed to assess quantitatively the area specific differences in immunoreactivity within and between areas of the retina in the control mice at P536 and *rd1* at P150, P300, and P536, in confocal images. Immunolabeling of cell markers was assessed from microscope images in a using a sliding window analysis in ImageJ (version 1.53n; provided in the public domain by the National Institutes of Health, Bethesda, MD, USA)^1^ and a custom MATLAB^®^ (R2020b, v9.9.0, Mathworks, Natrick, MA, USA) script that utilized the Image Processing Toolbox.

The window width was set to the pixel equivalent of 25 μm, translated from the scale bar on each image, and only pixels within the region of the neural retina were counted in the window. The neural retina in each window was defined as the pixels contained between the inner limiting membrane to the outermost part of the neural retina. Due to degeneration in rd1 mice, this outer border was manually delineated by user tracing of the retina within the image. As window width was constant, total pixel count within a given window was a function of retinal thickness.

To quantify an immunolabeled cell marker within a given window, images were separated into RGB channels and then individual pixels (P) were thresholded based on their respective pixel values, P_*PV*_ by the criterion


$$P_{T}=\left\{ \begin{array}{cc} 1, & i\,f\,P_{P\,V}>2\times M\,I_{P\,V}\\ 0, & i\,f\,P_{P\,V}\leq 2\times M\,I_{P\,V} \end{array}\right.$$

where P_T_ is the binary thresholded pixel value and MI_PV_ is the mean pixel value of the image for their respective channel and immunolabel. In short, individual pixels were considered above threshold if their value was greater than twice that of the relevant mean pixel value. The positive pixel ratio, PP_ratio_ of each window for each channel or immunolabel was then determined by


$$P\,P_{r\,a\,t\,i\,o}=\frac{\sum_{i=1}^{n}P_{T}}{n}$$

where *n* is the total number of pixels in the window counted as neural retina. In short, the positive pixel ratio revealed the proportion of pixels above threshold to the total number of pixels of the retina within the window, thus normalizing the positive pixel count to the thickness of the neural retina. The analysis was repeated across 25 μm windows across the whole length of the retina for each immunolabel in each microscope image using a window step size of 5 μm. In total, sliding window analysis was conducted on 37 images spanning 24,005 μm retinal length for control mice (*n* = 5 at P536), 90 images spanning 57,540 μm retinal length for rd1 mice at P150 (*n* = 5), 80 images spanning 51,050 μm retinal length for rd1 mice at P300 (*n* = 4) and 102 images spanning 58,435 μm of retina for rd1 mice at P536 (*n* = 6).

#### 2.3.2. Defining areas of normal and low glutamine synthetase immunolabeling

After analyzing the GS immunolabeling for all 24,005 μm of control retinae, the GS labeling of an Area (A) of rd1 retina was defined as either normal (GS_norm_) or low (GS_low_) utilizing the mean and standard deviations obtained from the control using the formula


$$A\,\overset{d\,e\,f}{=}\left\{ \begin{array}{cc} G\,S_{l\,o\,w}, & i\,f\,P\,P_{r\,a\,t\,i\,o}<M_{P\,P_{r\,a\,t\,i\,o}}-1.96\times S_{P\,P_{r\,a\,t\,i\,o}}\\ G\,S_{n\,o\,r\,m}, & i\,f\,P\,P_{r\,a\,t\,i\,o}\geq M_{P\,P_{r\,a\,t\,i\,o}}-1.96\times S_{P\,P_{r\,a\,t\,i\,o}} \end{array}\right.$$

where M_PPratio_ is the mean positive pixel ratio of the GS immunolabel for the control retina and S_PPratio_ is the standard deviation of the positive pixel ratio of the GS immunolabel for the control. In short, an area of rd1 retina was considered to have low GS immunolabeling if the proportion of GS pixels above threshold relative to total retina was less than the mean proportion of GS pixels relative to total retina by approximately two standard deviations or more.

#### 2.3.3. Defining colocalization of cell markers

**FIGURE 1:**
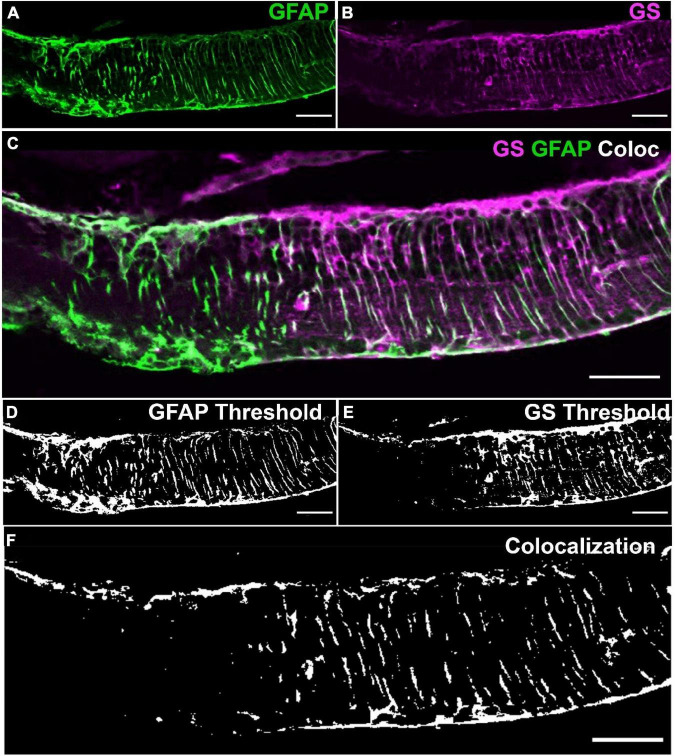
Method for defining colocalization of immunoreactivity. **(A)** Retinal tissue was double labeled with glutamine synthetase (GS; magenta) and **(B)** glial fibrillary acidic protein (GFAP; green). **(C)** Merged channels by merging GS and GFAP from images **(A,B)**. **(D,E)** Images **(A,B)** were thresholded with respect to the images’ mean pixel values to get binary maps of positive pixels. **(F)** The binary maps were then joined into a binary map where if and only if a pixel was positive for both GS and GFAP it was considered positive for colocalization of GS and GFAP. Note the similarities between the faint white in **(C)** and the binary map in **(F)**. Scale bar is 50 μm.

Pixel colocalization between any two immunolabels (for example labels A and B; Figures 1A, B and colocalisation in Figure 1C) in a retinal image was defined as a binary zero or one for each pixel within a retinal confocal image depending on whether its corresponding pixel values were over a set threshold (Figures 1D, E) such that


$$P_{C}=\left\{ \begin{array}{cc} 1, & i\,f\,P_{A_{P\,V}}>2\times M\,I_{A_{P\,V}}\,\wedge \,P_{B_{P\,V}}>2\times M\,I_{B_{P\,V}}\\ 0, & i\,f\,P_{A_{P\,V}}\leq 2\times M\,I_{A_{P\,V}}\vee P_{B_{P\,V}}\leq 2\times M\,I_{B_{P\,V}} \end{array}\right.$$

Where P_C_ is the thresholded co-localization pixel, P_APV_ and P_BPV_ are the individually thresholded pixel values for immunolabels A and B, respectively, and MI_APV_ and MI_BPV_ are the mean pixel values in the image for immunolabels A and B, respectively (Figure 1F). In short, if a pixel in a two-channel image matrix was positive for both A and B it was a site of colocalization. Colocalization ratio was then quantified as pixel values over threshold for both immunolabels divided by the total number of pixels of the respective area. All colocalization results were normalized to normal GS areas within the same retinal image for their relative assessment between images. For the assessment of colocalization between GS and GFAP, we assessed 6,698 μm of GFAP labeled rd1 (P536; *n* = 5) retina (see Table 1).

#### 2.3.4. Assessment of areas of normal and low glutamine synthetase immunolabeling

A total of 24,675 μm of rd1 retina (*n* = minimum 5 per age) was stained with DAPI for nuclear labeling. All positive pixel ratios, except GS, were normalized to the mean positive pixel ratios of normal GS areas within each retinal image to allow consistent, relative assessment of differences in other immunolabels between areas of normal vs. low GS areas within a retinal image. For the majority of cell markers, only retina at P536 were assessed as this was the only age that demonstrated numerous and long areas of low glutamine synthetase immunoreactivity that could be reliably quantified.

### 2.4. Statistical analysis

All variables are expressed as mean ± standard error. Data was analysed using the two-sample *t*-test and one way analysis of variance (ANOVA), with an α of 0.05. For *t*-tests with multiple numbers of tests the α was adjusted to the Bonferroni adjusted α (α_*B*_) of 0.0125 and 0.0167, when the number of tests were 4 and 3, respectively. For multiple groups a Tukey-Kramer *post-hoc* test was performed. Statistical analyses were performed using MATLAB^®^ (Mathworks, Natrick, MA, USA) v9.9.0.1570001 (R2020b) and the Statistics and Machine Learning Toolbox v12.0.

## 3. Results

### 3.1. Glutamine synthetase immunoreactivity is reduced in the rd1 mouse as a function of age

**FIGURE 2:**
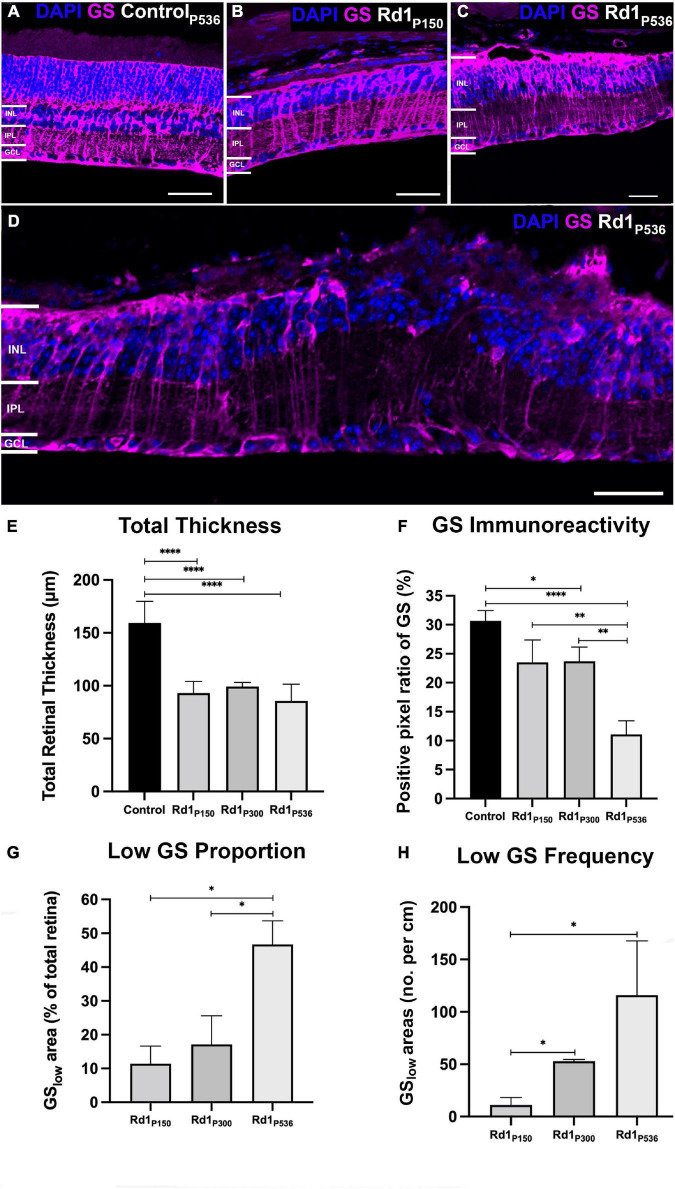
Glutamine synthetase immunoreactivity as a function of age in the rd1 retina. Representative images from a **(A)** healthy control retina at P536, and rd1 retina at **(B)** P150, **(C)** P300 and **(D)** P536 labeled for glutamine synthetase (GS; red) and 4’,6-diamidino-2-phenylindole (DAPI; blue). Scale bar is 50 μm. **(E)** Quantification of total retinal thickness confirming loss in the rd1 retina at all time points compared to control (*n* = 5, 5, 4, and 6 mice; mean ± SEM = 170.0 ± 7.4, 95.1 ± 4.7, 98.0 ± 1.4, and 84.3 ± 4.1; for C57Bl/6, rd1 P150, P300, P536, respectively). **(F)** Quantification of positive pixel ratio for GS demonstrating gradual loss in the rd1 retina relative to control (*n* = 5, 5, 4, and 6 mice; mean ± SEM = 31.6 ± 2.5, 24.2 ± 3.0, 22.7 ± 2.7, and 12.2 ± 1.6%; for C57Bl/6, rd1 P150, P300, P536, respectively). **(G)** Quantification of total amount of GS_low_ areas in rd1 retina defined as area where GS positive pixel count was below the mean-1.96 × SD of the control retina (*n* = 5, 4, and 6 mice; mean ± SEM = 10.4 ± 11.1, 17.7 ± 8.3, and 51.9 ± 8.2%; for rd1 P150, P300, P536, respectively). **(H)** Quantification of number of GS_low_ areas in the rd1 retina per cm (*n* = 4, 2, and 5 mice; mean ± SEM = 10.6 ± 6.5, 48.2 ± 4.5, and 139.4 ± 43.7 patches/cm; for rd1 P150, P300, P536, respectively). All data is presented as mean ± SEM. Statistical comparisons were performed *via t*-test with α_B_ = 0.0125 for **(E,F)** and α_B_ = 0.0167 for **(G,H)** and. All significant *p*-values are annotated on graphs. INL, inner nuclear layer; IPL, inner plexiform layer; GCL, ganglion cell layer; *p ≤ 0.05; ^**^p ≤ 0.01; ^****^p ≤ 0.0001.

The rd1 retinae was assessed at post-natal days P150, P300, and P536 and compared to control tissue at post-natal day P536 (Figures 2A–D). Total thickness of the rd1 retina was significantly reduced at all time points compared to control (*t*-test, all *p* < 0.0001, α_B_ = 0.0125; Figure 2E). However, there was no significant difference in thickness between each of the rd1 age groups (*t*-test, *p* > 0.0125, α_B_ = 0.0125; Tukey-Kramer, *p* > 0.05), though some regional thickening was qualitatively observed in low GS regions (Figure 2D). Thickness of individual retinal layers was not explored due to nuclear migration which prohibited accurate manual layer segmentation.

Glutamine Synthetase immunoreactivity was not significantly different between the rd1 P150 and control retinae (*t*-test, *p* = 0.73; Figures 2A, B, F). At P300, there was evidence of areas of the rd1 retina where GS immunoreactivity was absent or low compared to control retinae (Figure 2C). Quantification confirmed this with a 28% loss in GS positive pixel ratio in the rd1 retina at P300 relative to control, however, this was not significant following Bonferroni adjusted α (*t*-test, *p* < 0.05, α_B_ = 0.0125; Figure 2F). At P536, GS immunoreactivity further decreased (Figure 2D) with a significant 61% loss in GS positive pixel ratio relative to control (*t*-test, *p* < 0.0001, α_B_ = 0.0125; Tukey-Kramer, *p* < 0.0001). The standard deviation for the Positive Pixel ratio of GS in the control was 0.565. For the rd1 at P150, P300, and P536 it was 0.0666, 0.0537, and 0.0400, respectively, such that raw variability was similar for all groups.

Areas of low or absent GS immunoreactivity in the rd1 retina increased in length and number as a function of age. Specifically, areas of low GS immunoreactivity grew from 10.4 ± 5.0% of the total retina at P150 to 51.9 ± 8.2% by P536 (*t*-test, *p* < 0.0167, α_B_ = 0.0167; Turkey-Kramer, *p* < 0.01; Figure 2G). Similarly, the number of individual areas of low GS immunoreactivity per unit length of retina significantly increased 13-fold from P150 to P536 (*t*-test, *p* < 0.0167, α_B_ = 0.0167; Tukey-Kramer, *p* > 0.05; Figure 2H).

### 3.2. Müller cells are conserved in areas of low glutamine synthetase immunoreactivity

**FIGURE 3:**
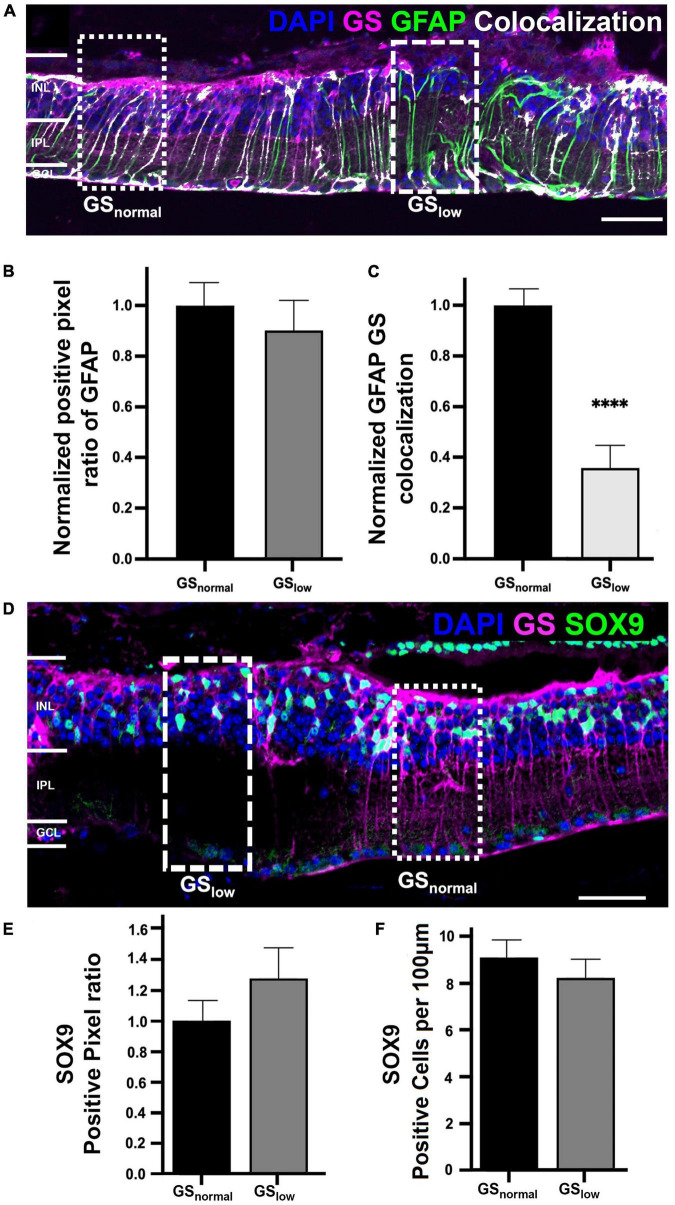
Representative images of the rd1 retina at P536 labeled for **(A)** glutamine synthetase (GS; magenta), glial fibrillary acidic protein (GFAP; green), GFAP colocalized with GS (white), and 4’,6-diamidino-2-phenylindole (DAPI) counterstaining (blue). Dotted boxes indicated a representative area of normal and low GS immunoreactivity, respectively. **(B)** Graph depicting normalized GFAP immunoreactivity in normal versus low GS immunoreactivity areas (*n* = 7 eyes; GS_norm_ = 1 ± 0.10, GS_low_ = 0.91 ± 0.12; *p* = 0.49). **(C)** Graph depicting the normalized colocalization of pixels for GS and GFAP (*n* = 7 eyes; GS_norm_ = 1 ± 0.07, GS_low_ = 0.35 ± 0.10). **(D)** Representative image of the rd1 retina at P536 labeled for glutamine synthetase (GS; magenta), Sox9 (green), and DAPI (blue). **(E)** Graph depicting normalized Sox9 immunoreactivity in normal versus low GS immunoreactivity areas (*n* = 5 eyes; GS_norm_ = 1 ± 0.13, GS_low_ = 1.27 ± 0.19; *p* = 0.20). **(F)** Graph depicting the cell count of Sox9 positive cells per 100 μm of retina for areas of normal and low GS immunoreactivity (*n* = 5 eyes; GS_norm_ = 8.92 ± 0.81, GS_low_ = 8.11 ± 0.92 Sox9 positive cells/100 μm; *p* = 0.52). All GS_low_ pixel ratio data is presented as mean ± SEM. Statistical comparisons were performed *via t*-test with α = 0.05. Only significant *p*-values are annotated on graphs, all other *p*-values are noted in this legend. Scale bar is 50 μm; GS_normal_, area of normal GS expression; GS_low_, area of low GS expression; INL, inner nuclear layer; IPL, inner plexiform layer; GCL, ganglion cell layer; ^****^p ≤ 0.0001.

To determine if loss of GS immunoreactivity reflected loss of Müller cells, we assessed immunoreactivity of two alternative Müller cell markers, glial fibrillary acidic protein (GFAP) and Sox9. Despite the observation of numerous areas of low GS immunoreactivity in rd1 retina at P536 (Figure 3A), GFAP immunoreactivity was present throughout the retina and followed the Müller cell trunk, spanning the entire retinal thickness. In areas of normal GS immunoreactivity, GFAP was co-localized with GS suggesting both markers were likely reflective of Müller cell presence (Figure 3A). However, in areas of low GS immunoreactivity, the colocalization was reduced by over 50% (*t*-test, *p* < 0.0001; Figure 3C). When quantified, there was no significant difference in GFAP immunolabeling in areas of low GS immunoreactivity compared to those with normal GS immunoreactivity (*t*-test, *p* = 0.49; Figure 3B) which suggests that Müller cells were still present in areas of low GS immunoreactivity.

Similarly, Sox9 immunoreactivity was present throughout the rd1 retina at P536 and in areas where normal GS immunoreactivity was present, Sox9 was assessed in normal and low GS areas (Figures 3D–F). When quantified, there was no significant difference in Sox9 immunolabeling in areas of low GS immunoreactivity compared to normal GS immunoreactivity in terms of positive pixel ratio (*t*-test, *p* = 0.20; Figure 3E), nor cell count (*t*-test, *p* = 0.52; Figure 3F). This further supports the notion that Müller cells were still present in areas of low GS immunoreactivity.

### 3.3. Loss of GS immunoreactivity disproportionately affects neuronal populations

Glutamine synthetase contributes to a major function of Müller cells: the clearance of extracellular glutamate to maintain retinal neurons and their microenvironment (Matsui et al., 1999; Bringmann et al., 2006; Kalloniatis et al., 2013). Thus, localized loss of GS in the rd1 retina could significantly affect surrounding neural tissue through elevated extracellular glutamate levels (Robin and Kalloniatis, 1992; Lieth et al., 1998; Dkhissi et al., 1999; Delyfer et al., 2005). To assess this, we analyzed major retinal neural populations within areas of low GS immunoreactivity using established cell markers (Table 1) and compared them to normal areas of GS expression in the rd1 retina at P536.

#### 3.3.1. Bipolar cell marker immunoreactivity is reduced in areas of low GS immunoreactivity

**FIGURE 4:**
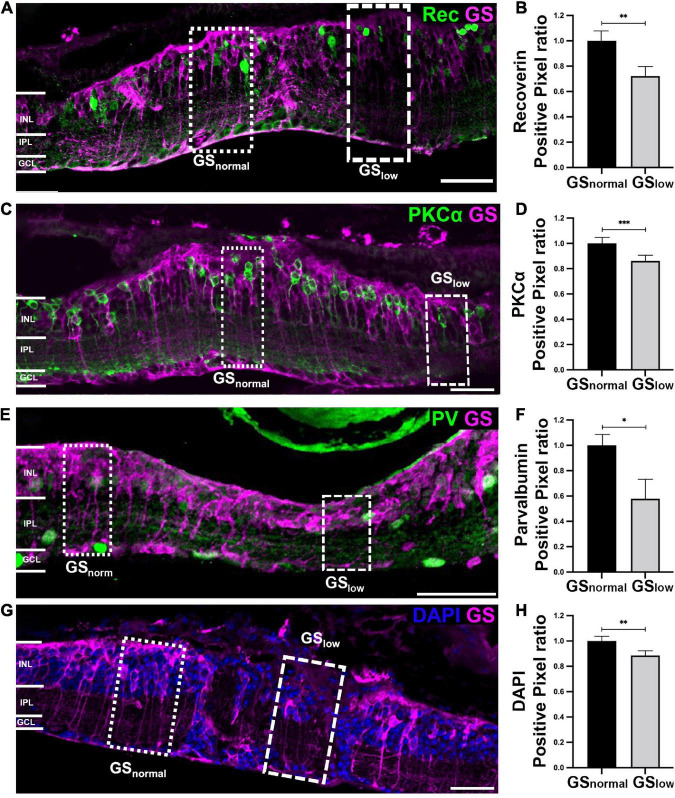
Representative images of the rd1 retina at P536 labeled for glutamine synthetase (GS; magenta) and **(A)** Recoverin (green), **(C)** PKCα (green), **(E)** Parvalbumin (green), or **(G)** 4’,6-diamidino-2-phenylindole (DAPI) (blue). Scale bar is 50 μm. Graphs on the right show normalized **(B)** Recoverin (*n* = 11 eyes; GS_norm_ = 1 ± 0.09; GS_low_ = 0.71 ± 0.08), **(D)** PKCα (*n* = 11 eyes; GS_norm_ = 1 ± 0.04; GS_low_ = 0.87 ± 0.03), **(F)** Parvalbumin (*n* = 8 eyes; GS_norm_ = 1 ± 0.09; GS_low_ = 0.62 ± 0.13), and **(H)** DAPI (*n* = 7 eyes; GS_norm_ = 1 ± 0.03; GS_low_ = 0.88 ± 0.03) positive pixels within areas of normal versus low GS immunoreactivity. All GS_low_ data is presented as mean ± SEM. Statistical comparisons were performed *via t*-test with α = 0.05. All significant *p*-values are annotated on graphs. GS_normal_, area of normal GS expression; GS_low_, area of low GS expression; INL, inner nuclear layer; IPL, inner plexiform layer; GCL, ganglion cell layer; *p ≤ 0.05; ^**^p ≤ 0.01; ^***^p ≤ 0.001.

Bipolar cells were assessed using cell markers against Recoverin (labels cone bipolar cell type 2 and type 8), protein kinase C-alpha (labels rod bipolar cells; PKC-α) and Islet-1 (labels all ON bipolar cells). Recoverin immunoreactivity was significantly decreased by 30% in areas of low GS immunoreactivity compared to normal GS immunoreactivity (*t*-test, *p* < 0.01; Figures 4A, B). A manual count of Recoverin labeled cells supported those findings (GS_normal_, 2.73 ± 0.31 Recoverin positive cells/100 μm; GS_low_, 1.57 ± 0.30 Recoverin positive cells/100 μm, *t*-test, *p* < 0.05). Similarly, PKC-α immunoreactivity was significantly decreased by 13% in areas of low versus normal GS immunoreactivity in the rd1 retina (*t*-test, *p* < 0.001; Figures 4C, D). There was a 27% decrease in Islet-1 immunoreactivity, however, this difference was not significant (*t*-test, *p* = 0.099; Supplementary Figures 1A, B).

#### 3.3.2. Select amacrine cell markers are reduced in areas of low GS immunoreactivity

Changes in GS immunoreactivity also impacted certain populations of amacrine cells, specifically AII amacrine cells based on a significant 40% reduction in Parvalbumin immunoreactivity in areas of low GS immunoreactivity, relative to normal GS immunoreactive areas (*t*-test, *p* < 0.05; Figures 4A, B). Assessment of horizontal cells and a subpopulation of amacrine cells *via* the cell marker Calbindin revealed no significant difference between areas of normal and low GS immunoreactivity (*t*-test, *p* = 0.082; Supplementary Figures 1C, D). Calretinin, which stains amacrine cells in the inner nuclear layer as well as wide-field amacrine cells and some ganglion cells in the ganglion cell layer, was also not significantly different between the two areas (*t*-test, *p* = 0.058; Supplementary Figures 1E, F). Finally, there was no significant difference in Glutamic acid decarboxylase 67 (GAD67) immunoreactivity in areas of low versus normal GS immunoreactivity, suggesting alterations in GS immunoreactivity did not affect GABAergic amacrine cells (*t*-test, *p* = 0.955; Supplementary Figures 1G, H).

#### 3.3.3. Nuclei based immunoreactivity is reduced in areas of low GS immunoreactivity

Finally, we explored if the loss of Recoverin, PKCα and PV cell marker immunoreactivity in areas of low GS immunoreactivity reflected the loss of the specific neuron population or simply loss of the cell marker. By assessing DAPI we found that areas of low GS immunoreactivity had a significant 12% decrease in total DAPI immunoreactivity relative to areas of normal GS immunoreactivity (*p* < 0.01; Figures 4G, H). This suggests that the loss of neurochemical markers noted above was due to loss of somata rather than a reduction in immunoreactivity alone.

## 4. Discussion

Diseases that result in photoreceptor death such as RP lead to aberrant functional and anatomical changes that follow a stage wise progression. The progression of these stages differs between the neural and glial population in the retina. While previous work indicates that GS expression remains stable in wildtype mice central nervous system for up to 18 months, (Olabarria et al., 2011), our results show that GS immunoreactivity is progressively diminished in the rd1 retina as areas of low GS both increase in number and size as the disease progresses.

Unaltered immunoreactivity of GFAP and Sox9 between regions of normal and low GS immunoreactivity in the rd1 retina suggest Müller cells were still however, present in these regions. GS immunoreactivity only affected some subpopulations of retinal neurons with reduction in bipolar cell marker immunoreactivity and AII amacrine cell marker immunoreactivity. Reduced DAPI labeling within low GS regions further suggested loss of cell marker immunoreactivity was at least in part attributed cell loss rather than a reduction in immunoreactivity alone.

### 4.1. Reduced GS immunoreactivity likely reflects Müller cell dysfunction, not absence in the rd1 retina

This study found GS immunoreactivity was reduced in the rd1 retina as a function of disease progress. Reduction in GS expression has been previously noted following photoreceptor degeneration (Härtig et al., 1995; Greferath et al., 2015; Jones et al., 2016; Pfeiffer et al., 2016, 2020b), retinal injury (Grosche et al., 1995), and retinal detachment (Lewis et al., 1989) and is possibly a consequence of the glutamate-release from dying photoreceptors. We qualitatively observed regional thickening of the retina in areas of low GS immunoreactivity however, quantitatively, no significant change in the overall thickness of the rd1 retina was found from P150 to P536, which suggested that strategies to minimize cell death in the INL may be of little value for RP treatments aimed at these stages of degeneration. Loss of GS immunoreactivity in the rd1 mouse appeared to reflect Müller cell dysfunction rather than Müller cell loss as immunoreactivity of other Müller cell markers, notably GFAP and Sox9, were not lost across the rd1 retina. Greferath et al. (2015) reported similar findings in the rd1-FTL retina. The exact process of dysfunction in non GS immunoreactive Müller cells is unclear however, it has been postulated that absence of this well-established metabolic pathway leads to “unmasking” of alternative, less energetically favorable metabolic pathways that attempt to continue the glutamate-glutamine cycle (Pfeiffer et al., 2020a).

Loss of GS immunoreactivity was highly variable across the retina with areas of low GS immunoreactivity immediately flanked by areas of GS immunoreactivity that were comparable to age-matched control retinae. While similar observations were made in the human RP, the P34TL rabbit and the rd1-FTL mouse (Jones et al., 2016; Pfeiffer et al., 2016, Pfeiffer et al., 2020b; Greferath et al., 2015), to our knowledge, this is the first attempt to specifically quantify this variable GS immunoreactivity loss in late-stage retinal degeneration of the rd1 mouse. Our analysis found that both the number and size of low GS immunoreactive areas increased significantly as a function of age and that by P536, 50% of the total retinal area demonstrated low GS immunoreactivity.

A possible reason for “patchy” loss of GS immunoreactivity in the rd1 retina could be the nature of photoreceptor loss which is also variable. This is supported by Greferath et al. (2015) who demonstrated that normal glutamine synthetase immunoreactivity was only maintained in the rd1-FTL retina in areas with remnant cone photoreceptor terminals. While we did not investigate GS immunoreactivity relative to any synaptic markers in this study, previous work indicates up to 5% of cone photoreceptors remain in the rd1 retina by P536 and therefore a spatial association between GS immunoreactivity and photoreceptor degeneration could potentially exist up to this stage.

Variable photoreceptor degeneration may have induced GS loss through uneven glutamate release and subsequent response from Müller cells (Härtig et al., 1995). However, Jones et al. (2003) noted that, in some instances, glutamate levels are elevated in Müller cells of degenerate retinae which would suggest greater demand for GS. Alternatively, release of basic fibroblast growth factor (bFGF) in response to neuronal damage (Gao and Hollyfield, 1995, 1996; Cao et al., 1997) could decrease GS expression in Müller cells through activation of the c-Jun signaling pathway (Kruchkova et al., 2001). Disruption of GS expression has been shown to lead to a breakdown of the blood-retinal barrier (Shen et al., 2010) which could lead to further cell death and release of bFGF, thus creating a feedback loop that contributes to the spread of low GS areas with disease progression.

### 4.2. Areas of low GS immunoreactivity display advanced features of degeneration

Greferath et al. (2015) found that in the rd1 FTL mouse, *c-fos* was increased within areas of low GS immunoreactivity which suggested greater neural activity, cell death, and/or plasticity (Rich et al., 1997). However, no individual neuronal populations have been previously analyzed in areas of low GS immunoreactivity. In this study, we found the immunoreactivity of bipolar cell markers Recoverin and PKCα and the AII amacrine cell marker Parvalbumin were significantly reduced in areas of low GS immunoreactivity. Nuclei labeling was also significantly reduced in areas of low GS immunoreactivity suggesting that loss of these markers at least partly reflected cell loss rather than solely a reduction in cell marker immunoreactivity. Alternatively, reduction in marker immunoreactivity and cell nuclei number in low GS immunoreactive areas could be a consequence of neuronal migration described in advanced retinal remodeling (Jones et al., 2003). The direction of cell migration in retinal degeneration has been previously described to follow along glial surfaces to ectopic sites, which is inconsistent with the decreased DAPI observed in low GS immunoreactive areas in this study. However, retinal migration is still poorly understood with a number of different migration patterns observed including evidence of neurons migrating out of the degenerating retina *via* the choroid (Sullivan et al., 2003).

The loss of PKCα and Parvalbumin immunoreactivity which labels rod bipolar cells and AII amacrine cells, respectively, suggests that inner retinal neurons involved in the rod-mediated pathway are significantly affected by low GS immunoreactivity. Reduction in Recoverin immunoreactivity may reflect this as well, as type 2 bipolar cells have a high degree of chemical synaptic contacts with AII amacrine cells (Tsukamoto and Omi, 2017). Greater impairment of the rod-mediated pathway also aligns with the model of degeneration as rod loss precedes cone death in the rd1 retina (Jiménez et al., 1996).

### 4.3. Retinal degeneration as a feedback loop

Based on the results of this study we postulate that following photoreceptor death, areas of low GS develop. These areas become more numerous and expand, which likely leads to excess extracellular glutamate. As a result, local populations of glutamatergic neurons to become hyperactive and possibly excitotoxic. This could explain the increased c-fos labeling in the rd1-FTL mouse in regions of GS loss (Greferath et al., 2015) and the reduction in amacrine and bipolar cell marker immunoreactivity seen in this study. Altered glutamate homeostasis could also shed light on the canonical glutamate receptor function and class switching seen in late-stage retinal degeneration and remodeling (Puthussery et al., 2009; Jones et al., 2016). Finally, death or dysfunction of select amacrine and bipolar cell populations within areas of low GS could lead to the release of exogenous bFGF, which would feedback to further decreasing GS expression and exacerbating remodeling. Future work is needed to characterize Müller cells and retinal neuron subtypes within these regions as it will help build our understanding of these glia-neuronal alterations reported in late-stage degeneration.

### 4.4. Limitations

Due to the late stage of degeneration, we could not easily make a distinction between the inner nuclear layer, inner plexiform layer, and ganglion cell layer in the rd1 retina and thus assessed all retinal layers as one. As a result, we are limited in the conclusions we can make with regard to retinal migration. We were also unable to account for any lateral displacement meaning neurons which originate from low GS immunoreactive areas but migrate to normal GS immunoreactive areas were counted as the later. We believe this effect however, was minimal based on regions of low GS immunoreactivity being large in the P536 retina and very few quantifying markers being at the borders of low and normal GS areas.

Another by-product is that small changes in immunoreactivity in selected neuronal populations may have been masked by cell markers that label multiple cell subtypes. For example, we found no significant difference in Islet-1 immunoreactivity in areas of normal vs. low GS immunoreactivity despite evidence of rod bipolar cell loss through PKCα labeling. This may have been due to Islet-1 labeling of surviving amacrine cells which were found to be unaffected by GS immunoreactivity loss. Similarly, Calretinin immunoreactivity was not significantly altered between normal and low GS immunoreactive areas. However, this could have been due to labeling displaced amacrine cells and ganglion cells in the ganglion cell layer. Despite this our sliding window quantitative analysis ensured our whole retina analysis was a systematic and unbiased evaluation of immunoreactivity across the retina. Our analysis did not assess variability as a function of eccentricity as all samples were within 500 μm of the central retina. Future work with more specific cell markers could determine if other neuronal populations are affected by GS immunoreactivity changes.

## 5. Conclusion

Glutamine synthetase (GS) is lost in the rd1 retina as discrete regions that increased in both size and number as a function of age. This loss is not likely due to Müller cell loss as GFAP immunoreactivity was unaltered. Specific loss of neural macromolecular markers pertaining to specific amacrine and bipolar cell populations occurred in areas of low GS immunoreactivity and this was likely, in part, due to neuronal death based on decreased DAPI labeling. These data shed light on glia-neuronal alterations in late-stage degeneration and could provide insight for interventions to combat them.

## Data availability statement

The raw data supporting the conclusions of this article will be made available by the authors, without undue reservation.

## Ethics statement

This animal study was reviewed and approved by University of Melbourne and University of New South Wales Animal Ethics Committees.

## Author contributions

LN-S performed the tissue preparation, immunostaining, and imaging. HR performed the quantification, programming, and statistical analysis. EF supplied the mice. MS provided study funding in part. All authors contributed to the manuscript and approved the submitted version.

## Footnotes

1
http://imagej.nih.gov/ij/
