# Supplementary material for: Loss of Müller cell glutamine synthetase immunoreactivity is associated with neuronal changes in late-stage retinal degeneration
Source: Front Neuroanat. 2023 Mar 7;17:997722. doi: 10.3389/fnana.2023.997722 (PMC10029270; doi:10.3389/fnana.2023.997722)
Supplement: Supplementary file 1 [file Image_1.pdf]

Supplementary Material

1 Supplementary Figure

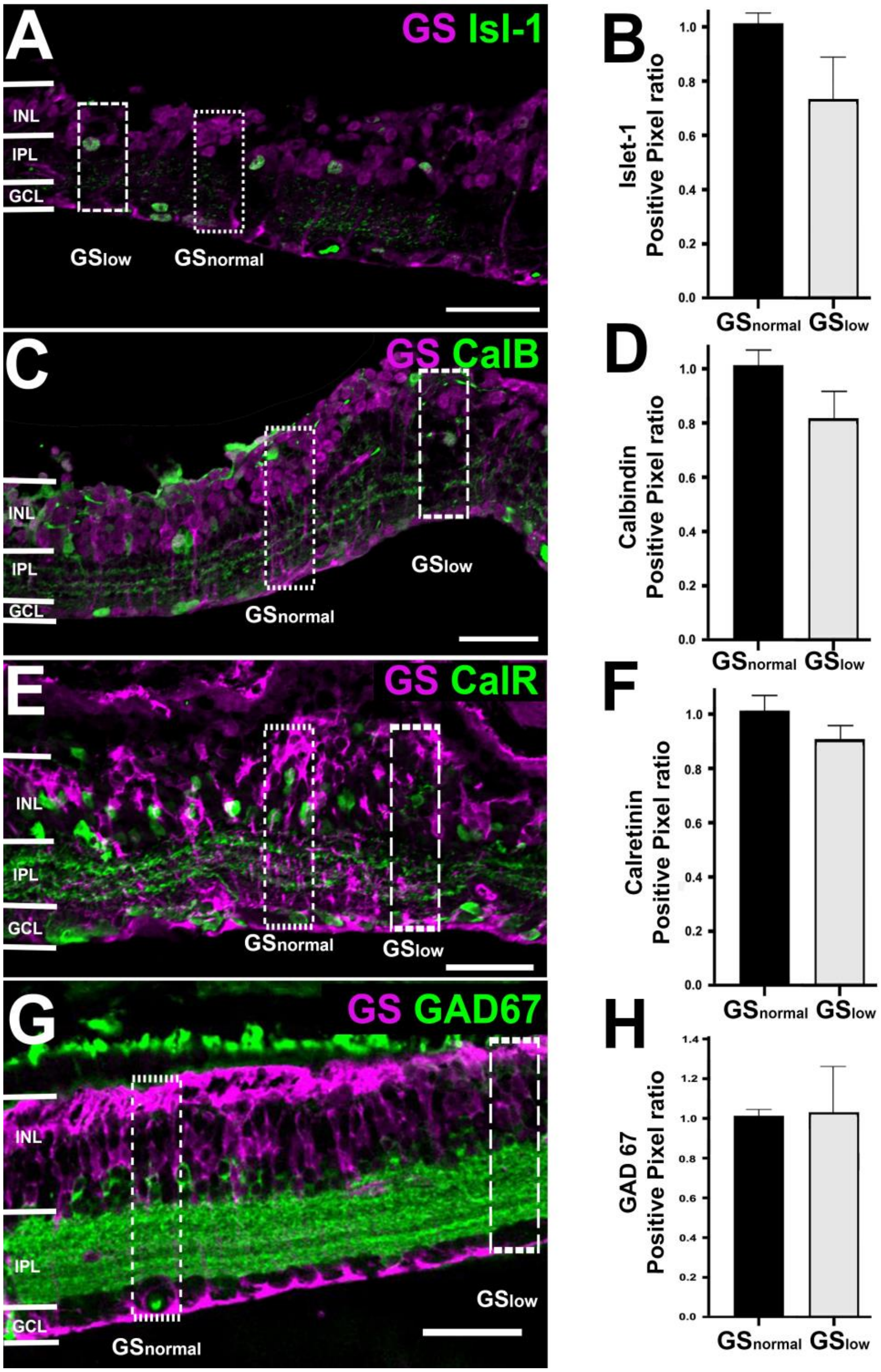

Supplementary Figure 1: Representative images of the rd1 retina at P536 labelled for glutamine synthetase (GS; magenta) and (A) Islet-1 (green), (C) Calbindin (green), (E) Calretinin (green), or (G) Glutamic acid decarboxylase 67 (GAD67; green). Dotted boxes indicated a representative area of normal and low GS immunoreactivity respectively. Scale bar is 50 µm. Graphs on the right show normalized (B) Islet-1 (n =8 eyes; GS_{norm} = 1±0.04; GS_{low} = 0.72 ±0.16; p = 0.098), (D) Calbindin (n =6 eyes; GS_{norm} = 1±0.06; GS_{low} = 0.80±0.10; p = 0.082), (F) Calretinin (n =11; GS_{norm} = 1±0.06; GS_{low} = 0.89±0.05; p = 0.058), or (H) Glutamic acid decarboxylase 67 (n =4 eyes; GS_{norm} = 1±0.03; GS_{low} = 1.01±0.23; p =0.95) positive pixels within areas of normal versus low GS immunoreactivity. All GS_{low} data is presented as mean ± SEM. Statistical comparisons were performed via t-test with $\alpha = 0.05$. Only significant p-values are annotated on graphs, all other values are noted in this legend. Abbreviations: Area of normal GS expression, GS_{normal}. Area of low GS expression, GS_{low}. Inner Nuclear Layer, INL; Inner Plexiform Layer, IPL; Ganglion Cell Layer, GCL; $p \leq 0.05$, *; $p \leq 0.01$, **; $p \leq 0.001$, ***; $p \leq 0.0001$, ****.
